# Epigenetic regulations in the IFNγ signalling pathway: IFNγ-mediated MHC class I upregulation on tumour cells is associated with DNA demethylation of antigen-presenting machinery genes

**DOI:** 10.18632/oncotarget.2222

**Published:** 2014-07-15

**Authors:** Veronika Vlková, Ivan Štěpánek, Veronika Hrušková, Filip Šenigl, Veronika Mayerová, Martin Šrámek, Jana Šímová, Jana Bieblová, Marie Indrová, Tomáš Hejhal, Nicolas Dérian, David Klatzmann, Adrien Six, Milan Reiniš

**Affiliations:** ^1^ Department of Tumour Immunology, Institute of Molecular Genetics, Academy of Sciences of the Czech Republic, v. v. i., Prague; ^2^ Department of Viral and Cellular Genetics, Institute of Molecular Genetics, Academy of Sciences of the Czech Republic, v. v. i., Prague; ^3^ Sorbonne Universités, UPMC Univ Paris 06, UMR 7211, Immunology-Immunopathology-Immunotherapy (I3) Paris, France; ^4^ CNRS, FRE 3632, Immunology-Immunopathology-Immunotherapy (I3), Paris, France; ^5^ INSERM, UMR_S 959, Immunology-Immunopathology-Immunotherapy (I3), Paris, France; ^6^ AP-HP, Hôpital Pitié-Salpêtrière, CIC-BTi Biotherapy & Département Hospitalo-Universitaire (DHU) Inflammation-Immunopathology-Biotherapy (i2B), Paris, France

**Keywords:** IFNγ signalling, DNA demethylation, 5-azacytidine, MHC class I downregulation, tumour immunology

## Abstract

Downregulation of MHC class I expression on tumour cells, a common mechanism by which tumour cells can escape from specific immune responses, can be associated with coordinated silencing of antigen-presenting machinery genes. The expression of these genes can be restored by IFNγ. In this study we documented association of DNA demethylation of selected antigen-presenting machinery genes located in the MHC genomic locus (*TAP-1, TAP-2, LMP-2, LMP-7*) upon IFNγ treatment with MHC class I upregulation on tumour cells in several MHC class I-deficient murine tumour cell lines (TC-1/A9, TRAMP-C2, MK16 and MC15). Our data also documented higher methylation levels in these genes in TC-1/A9 cells, as compared to their parental MHC class I-positive TC-1 cells. IFNγ-mediated DNA demethylation was relatively fast in comparison with demethylation induced by DNA methyltransferase inhibitor 5-azacytidine, and associated with increased histone H3 acetylation in the promoter regions of APM genes. Comparative transcriptome analysis in distinct MHC class I-deficient cell lines upon their treatment with either IFNγ or epigenetic agents revealed that a set of genes, significantly enriched for the antigen presentation pathway, was regulated in the same manner. Our data demonstrate that IFNγ acts as an epigenetic modifier when upregulating the expression of antigen-presenting machinery genes.

## INTRODUCTION

Epigenetic changes, such as aberrant DNA methylation, play important roles in carcinogenesis [1,2] and namely in the tumour cell escape from anti-tumour immune responses [3,4]. MHC class I downregulation on tumour cells represents a frequent mechanism by which tumour cells can escape from anti-tumour specific immunity [5-8]. The molecular defects responsible for impaired MHC class I expression on the tumour cell surface can be either irreversible (“hard”) or reversible (“soft”) [9]. The latter can be associated with coordinated silencing of antigen-presenting machinery (APM) genes in tumour cells [10,11] and the expression of these genes can be restored by IFNγ [10,12].

An important task is whether epigenetic events, such as changes in DNA methylation, take place in concerted APM gene silencing and IFNγ-induced restoration of their expression. Evidence that epigenetic mechanisms are important in MHC class I downregulation in APM-deficient tumours and its IFNγ-mediated induction was brought by Setiadi et al. [13]. The lack of *TAP-1* transcription in TAP-deficient cells was associated with low levels of recruitment of histone acetyltransferase CBP (CREB-binding protein) to the *TAP-1* promoter. IFNγ-mediated MHC class I expression corresponded to upregulation of the *TAP-1* expression by increasing histone H3 acetylation at the *TAP-1* promoter locus. Another study documented higher-order chromatin remodelling and subsequent histone hyperacetylation of the human MHC locus upon IFNγ-mediated activation of the JAK/STAT signalling pathway [14].

We, as well as other laboratories, have previously documented that DNA methylation and histone acetylation might play a role in reversible MHC class I deficiency on the tumour cell surface, since it could be partially restored by the treatment with DNA methyltransferase or histone deacetylase inhibitors [15-17]. This increase was associated with elevated expression of antigen-presenting machinery genes, such as *TAP-1*, *TAP-2*, *LMP-2 (PSMB9), LMP-7 (PSMB8*), as well as with DNA demethylation of their corresponding regulatory sequences. We have also shown that chemotherapy of MHC class I-deficient tumours with 5-azacytidine (5AC) in mice increased the expression of the APM genes and associated MHC class I molecule cell surface expression and we have demonstrated 5AC additive effects against MHC class I-deficient tumours when combined with immunotherapy. Notably, the efficacy of this chemoimmunotherapy was partially dependent on the CD8^+^-mediated immune responses [18].

Unlike chromatin remodelling and histone acetylation dynamics, the changes in DNA methylation upon activation of the IFNγ signalling pathway have not been studied so far. Based on the fact that a set of the APM genes is upregulated by both IFNγ and DNA methyltransferase inhibitors, we have hypothesized that IFNγ-mediated re-activation of silenced APM and some other genes is also associated with their DNA demethylation. The objective of this study was to uncover the association of DNA methylation with IFNγ-mediated upregulation of genes encoding the components of APM in MHC class I-deficient murine tumour cell lines.

## RESULTS

### MHC class I molecule upregulation on tumour cells upon the IFNγ treatment

**Figure 1:**
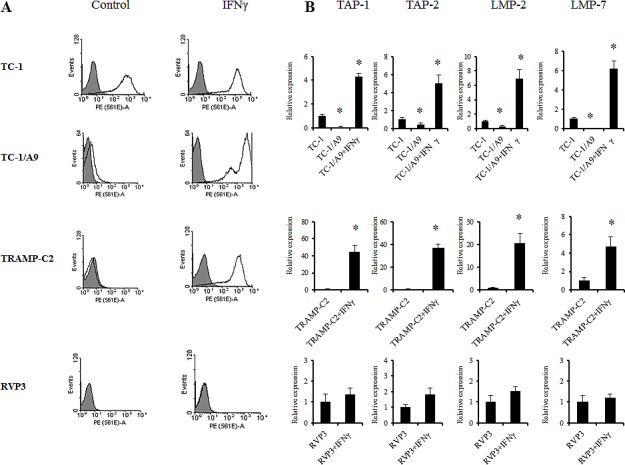
IFNγ upregulation of the cell-surface MHC class I expression cells is associated with APM gene expression in experimental tumour cells MHC class I expression (H-2D^b^ and H-2K^b^ together) was determined by the FACS analysis of control tumour cells and after the treatment with IFNγ. Representative results are presented. (A) Upregulation of APM genes in TC-1/A9 and TRAMP-C2 tumours after treatment with IFNγ. (B) Expression levels of selected APM genes in TC-1/A9 and TRAMP-C2 control tumour cells and after the treatment with IFNγ. As a negative control, MHC class I-deficient RVP-3 cell line that did not respond to the IFNγ treatment was used. TC-1 cells, a MHC class I-positive parental cell line to the TC-1/A9 cells, that also displayed higher APM expression levels compared to TC-1/A9 cells, served as a MHC class I-positive control. *denote significant changes (P<0.05 determined in Student's t-test) as compared to the values for untreated cells. Biological triplicates were used for the analysis. In all experiments, error bars show standard deviations. Relative expression numbers represent the percentage of the β-actin expression levels. The levels of relative gene expression were presented as fold changes compared to the levels found in control samples. Experiments were repeated three times with similar results.

First, we assessed the level of the MHC class I and APM molecule expression and its modulation by IFNγ on the selected tumour cells (Fig. 1a). MHC class I expression on tumour cells after IFNγ treatment was upregulated as compared to the tumour cells without treatment. As expected and as has been published previously [19,20], the MHC class I upregulation induced by IFNγ was associated with increased expression of APM genes (Fig. 1b). As a negative control, we used the MHC class I-deficient RVP-3 cell line that did not respond to the IFNγ treatment. TC-1 cells, an MHC class I-positive parental cell line to the TC-1/A9 cells, which also displayed higher APM expression levels, as compared to TC-1/A9 cells, served as an MHC class I-positive control.

### The IFNγ-mediated increase of MHC class I expression on tumour cells and APM gene machinery induction are associated with DNA demethylation of the corresponding APM gene regulatory sequences

**Figure 2:**
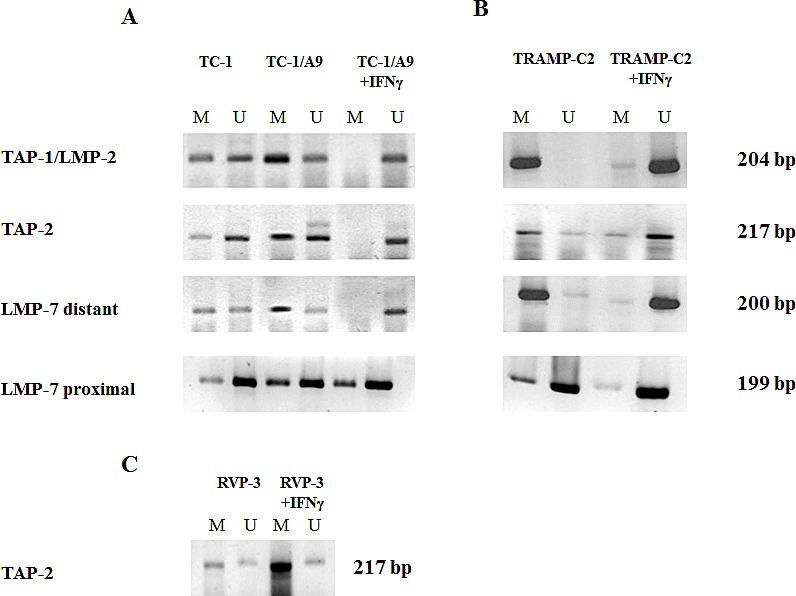
IFNγ stimulates DNA demethylation of the APM gene promoter regions DNA from tumour cell lines cultured in the absence or presence of IFNγ were bisulphite treated and subjected to MSP analysis of the *TAP-1/LMP-2, TAP-2* and *LMP-7* promoter sequences. Higher proportion of DNA methylation, as compared to TC-1 cells and DNA demethylation induced by IFNγ, is documented in TC-1/A9 cells (A). Similar results were obtained in TRAMP-C2 cells (B), while no effects were noticed in IFNγ-insensitive RVP-3 tumour cells (C). U = unmethylated primer, M = methylated. Experiments were repeated three times with similar results.

Enhanced APM gene expression in the MHC class I-deficient tumour cells was associated with DNA demethylation of the corresponding gene promoter regions determined by MSP (Fig. 2). We demonstrated DNA demethylation of the promoter sequences of selected antigen-presenting machinery genes (*TAP-1/LMP-2, TAP-2*) upon IFNγ treatment both in the MHC class I-deficient tumour cell line TC-1/A9 (Fig. 2a) and in the prostate cancer cell line TRAMP-C2 (Fig. 2b). For *LMP-7*, we did not see any dramatic changes in the MSP analysis targeting cytosines located at positions -186, -190 and -335 upstream from the LMP-7 transcription start (proximal primers). We therefore analysed CpGs in a more distant region covering CpGs at the positions -1219, -1233 and -1238 and -1087. In this region, we indeed noticed massive demethylation upon IFNγ treatment (distant primers). As a positive control, we used DNA from the MHC class I-positive TC-1 cell line (Fig. 2a), and as a negative control, we used MHC class I-deficient RVP-3 cell line, which did not respond to the IFNγ treatment (Fig. 2c). Comparative analysis of the TC-1 and TC-1/A9 cell lines demonstrated association of the cell surface MHC class I expression levels with DNA demethylation of the APM genes. No demethylation of the APM genes upon IFNγ treatment was seen in the RVP-3 cells.

**Figure 3:**
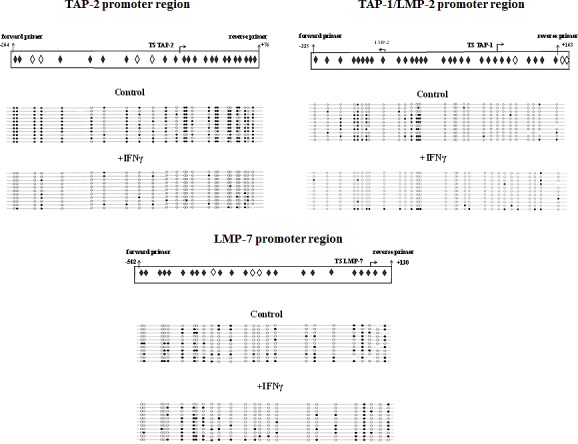
IFNγ-induced DNA demethylation of the *TAP-2* and *TAP-1/LMP-2* promoters in TC-1/A9 cells analysed by bisulphite sequencing DNA isolated from treated and control untreated TC-1/A9 cells was subjected to bisulphite conversion and cloned. Sequences from 11 clones from each sample are presented. After treatment with IFNγ, strong DNA demethylation of both the *TAP-2* and *TAP-1/LMP-2* gene promoter regions was observed. For LMP-7, we did not see any dramatic changes in bisulphite sequencing analysis targeting cytosines located at the positions -502 upstream to +130 downstream from the LMP-7 transcription start site. White and black circles indicate unmethylated and methylated CpGs, respectively. Rhombuses indicate the CpG islands that were investigated with bisulphite sequencing. White colour marks the CpG islands investigated with MSP. TS: transcription start.

Results from the MSP were confirmed by bisulphite sequencing using the TC-1/A9 cell line (Fig. 3). Again, strong DNA demethylation of both the *TAP-2* and *TAP-1/LMP-2* gene promoter regions was observed after the treatment with IFNγ. For LMP-7, we did not see any dramatic changes in a bisulphite sequencing analysis targeting cytosines located at the positions -502 upstream to +130 downstream from the LMP-7 transcription start site. This corresponds with the result from MSP analysis with LMP-7 proximal primers. Based on these results, we can suggest that the methylation status of the distant rather than proximal regulatory sites in the *LMP-7* region is crucial for their expression.

Both TC-1/A9 and TRAMP-C2 cells represent experimental models for virally transformed tumour cells that do not metastasize. We therefore analysed two more MHC class I-deficient tumour cell lines, metastatic HPV16 E6/E7-positive MK16 and the methylcholantrene-induced MC15 cells (Supplementary Figure 1). Similarly to the experiments with TC-1/A9 and TRAMP-C2 cells, association of the cell surface MHC class I expression levels with DNA demethylation of the APM genes was observed.

### DNA demethylation corresponds to the histone H3 acetylation levels

**Figure 4:**
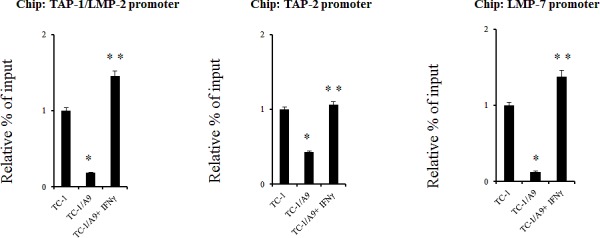
Histone H3 acetylation levels in the APM regulatory gene sequences in TC-1/A9 cells are lower than those in TC-1 cells, but can be increased by IFNγ ChIP analysis of chromatin from the *TAP-1/LMP-2, TAP-2,* and *LMP-7* promoter sequences isolated from control and treated TC-1/A9 cells demonstrates an increase in acetylated histone H3 (H3K18) after IFNγ treatment. Results were normalized to the levels of the relative input in TC-1 cells. Experiments were repeated five times with similar results. * denotes significant changes (P<0.05 determined in Student's t-test) as compared to the values from TC-1 cells. ** denotes significant changes (P<0.05 determined in Student's t-test) as compared to the values from TC-1/A9 cells.

ChIP assay was performed to determine whether the dose of IFNγ that was sufficient to reverse the methylation of the *TAP-1/LMP-2* bidirectional promoter region, as well as *LMP-7* and *TAP-2* promoter regions, was able to modify the histones associated with this promoter (Fig. 4). The assay demonstrated that histone H3 on lysine 18 was re-acetylated after IFNγ treatment in all three tested regions. Acetylated histone H3 was detected in untreated TC-1/A9 cells at a low level. The TC-1 cell line served as a positive control with high levels of acetylated histone H3 and, as expected, the acetylation levels were higher in untreated TC-1 cells than in untreated TC-1/A9 cells.

### Kinetics of the DNA demethylation

**Figure 5:**
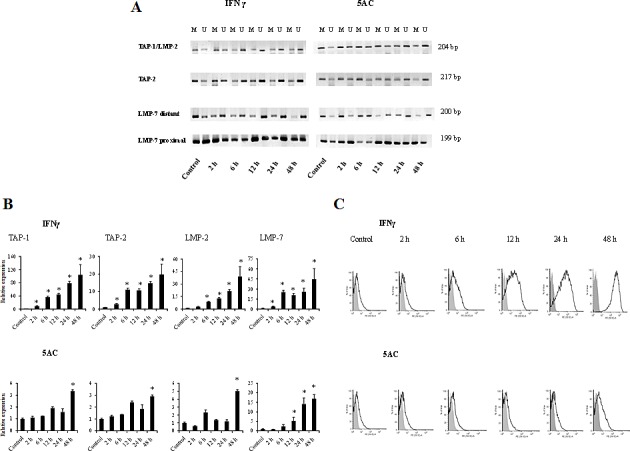
Comparative analysis of the kinetics of DNA demethylation of the APM genes induced by IFNγ or 5AC TC-1/A9 cells were cultured in the presence of either IFNγ or 5AC. For the indicated time periods, DNA samples were isolated, bisulphite treated and subjected to MSP analysis of the *TAP-1, TAP-2*, *LMP-2 & LMP-7* promoter sequences. U = unmethylated primer, M = methylated. In untreated cells, the core CpG island was highly methylated, and demethylation was detected within 2 hours after the IFNγ treatment, while nearly complete demethylation was evident by 6 hours (A). After 5AC treatment, strong demethylation was evident by 24 hours (A). The amount of 1 μg of RNA was reverse transcribed to cDNA and the PCR products were quantified. Upregulation of APM genes in TC-1/A9 cells after the treatment with IFNγ after 2 hours (A) and with 5AC after 48 hour (B). * denote significant changes (P<0.05 determined in Student's t-test) as compared to the values from untreated cells. Biological triplicates were used for the analysis. In all experiments, error bars show standard deviations. Relative expression numbers represent the percentage of the β-actin expression levels. The levels of relative gene expression were presented as fold changes compared to the levels found in control samples. MHC class I expression (H-2D^b^ and H-2K^b^ together) was determined by FACS analysis of the control tumour cells and after the treatment with IFNγ and 5AC. Representative results are presented (C). Experiments were repeated three times with similar results.

To examine the kinetics by which the APM promoter regions undergo IFNγ-mediated changes in DNA methylation, as compared to the effects of a DNA methyltransferase inhibitor, TC-1/A9 cells were treated with either IFNγ or 5AC for various time periods and then by sodium bisulphite conversion and MSP. In untreated cells, the core CpG island was highly methylated, and demethylation was noticed within 2 h after IFNγ treatment, while nearly maximal demethylation was evident by 6 h (Fig. 5). After 5AC treatment, strong demethylation was evident by 24 h. The kinetics of the 5AC-induced demethylation is in agreement with the fact that 5AC-induced demethylation required DNA replication. On the other hand, the kinetics of the IFNγ-mediated DNA demethylation suggests that DNA replication was not crucial.

### JAK/STAT inhibition studies

**Figure 6:**
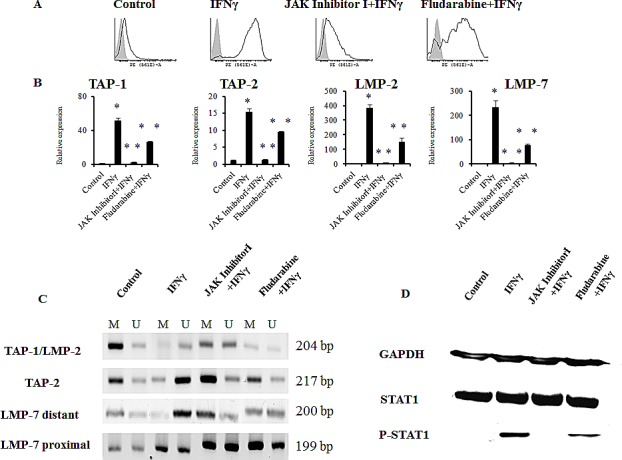
JAK/STAT inhibitors abrogated IFNγ-induced DNA demethylation of the APM gene promoters in TC-1/A9 cells Inhibitor of Janus kinases (JAK inhibitor 1), as well as STAT1 phosphorylation inhibitor fludarabine, blocked the IFNγ induction of the MHC class I cell-surface expression (A), as well as APM gene activation (B), and caused impaired demethylation of the corresponding gene promoter regions (C). Both inhibitors blocked IFNγ-induced STAT1 phosphorylation (D). The effect of fludarabine was much weaker, as compared to Janus kinase inhibitor 1. * denotes significant changes (P<0.05 determined in Student's t-test) as compared to the values from untreated control cells. ** denotes significant changes (P<0.05 determined in Student's t-test) as compared to the values from TC-1/A9 cells after IFNγ treatment. Biological triplicates were used for the analysis. In all experiments, error bars show standard deviations. Relative expression numbers represent the percentage of the β-actin expression levels. The levels of relative gene expression were presented as fold changes compared to the levels found in control samples. U = unmethylated primer, M = methylated primer. Experiments with JAK inhibitor 1 and fludarabine were repeated five and two times, respectively, with similar results.

The changes in gene expression by IFNγ involve transient increases in the activities of cellular protein tyrosine kinases, including the Janus kinases Jak1 and Jak2, leading to tyrosine phosphorylation of the transcription factor Stat1 [21]. To assess whether the JAK/STAT pathway was crucial for demethylation of the APM gene promoter regions in TC-1/A9 cells after IFNγ treatment, the impacts of an inhibitor of Janus kinases, as well as of STAT1 phosphorylation inhibitor fludarabine on IFNγ-induced demethylation were investigated (Fig. 6). Both inhibitors blocked IFNγ-induced STAT1 phosphorylation, although the effect of fludarabine was much weaker, as compared to Janus kinase inhibitor 1. Indeed, the inhibitor of Janus kinases caused impaired demethylation of the corresponding gene promoter regions, accompanied by decreased relative gene expression of selected APM genes, along with reduction of the MHC class I cell surface expression. Since JAK inhibitor 1 is not solely specific for JAK1, we also used fludarabine, which has been described as a STAT1 phosphorylation specific inhibitor. The effect of fludarabine corresponded to the limited STAT1 phosphorylation inhibition, as observed by the western blot analysis. Both MHC class I cell surface expression and APM gene expression induced by IFNγ was only partially inhibited and the MSP results suggested partial DNA demethylation. These results suggest that the classical IFNγ signalling pathway takes place in DNA demethylation, although other mechanisms cannot be excluded.

### Comparison of the impacts of IFNγ and epigenetic agent treatments on the transcriptome of the tumour cell lines

In these experiments, the aim was to analyse (i) the global impact of IFNγ compared to epigenetic treatment (namely DAC/TSA) on the gene expression in the TC-1/A9 cell line and (ii) whether IFNγ-induced APM genes can be upregulated upon epigenetic treatment. We compared transcriptome changes upon IFNγ *vs.* DAC/TSA treatments (compared to untreated control cells) of the TC-1/A9 cell line as compared to those of the IFNγ non-responding RVP3 cells. IFNγ-treated TC-1/A9 cells presented 105 significantly upregulated genes (FDR<0.01) and only two downregulated genes (*RIN2* and *LBH*). Treatment with DAC/TSA provided 2732 significantly upregulated and 2815 downregulated genes. This result can be explained by the fact that IFNγ targets specific genes, while DAC acts on the overall genome.

Out of the 105 upregulated genes in IFNγ-treated TC-1/A9 cells, we defined two gene sets: GS-IFN comprises the 73 genes that were specific to IFNγ treatment, when GS-COM comprises the 32 genes that are upregulated upon both treatments (GS-COM and GS-IFN gene lists are can be seen as Supplementary material, Table 1 and Table 2, respectively). Gene sets were annotated using IPA (Ingenuity®) for pathway enrichment (Benjamini-Hochberg controlled p-values). Both gene sets are significantly enriched for the “Cell Death Of Tumour Cell Lines” pathway (p-value = 1.1e-4 for GS-IFN and p-value = 5.64e-3 for GS-COM): 19 genes are specifically upregulated upon IFNγ treatment (*CASP4, CASP7, CLEC2D, DDX58, ENC1, FST, GDNF, IL15RA, IL7, IRAK2, IRF1, LGALS3BP, MLKL, SOCS3, STAT2, STAT3, TRIM21, UACA*, *UBA7*). Eight genes are upregulated in both treatments (*BID, CREM, EIF2AK2, FAS, HAP1, IDO1, STAT1*, *USP18*). As expected, some GS-IFN genes are related to the “Interferon Signaling” pathway (*IFIT3, IFI35, STAT2, IRF9, TAP1, IRF1*; p-value = 2.38e-7). Strikingly, GS-COM annotation identifies the “Antigen Presentation” pathway (*HLA-G, LMP-2 (PSMB9), HLA-B, LMP-7 (PSMB8), TAP2, TAPBP, HLA-E*; p-value = 1.23e-11).

Consistent results were obtained when comparing the impact of IFNγ on TC-1/A9 cells (IFNγ and DAC/TSA sensitive) and DAC/TSA effects on the RVP3 cell line (IFNγ resistant though DAC/TSA sensitive). The 40 upregulated genes in both conditions show enrichment for the “Antigen Presentation” pathway (*HLA-B, HLA-E, HLA-G, NLRC5, TAP2, TAPBP*; p-value=1.6e-8) and the 65 IFNγ-specific gene set is also enriched for the “Interferon Signaling” pathway (*IFIT3, IRF1, IRF9, LMP-7 (PSMB8), STAT2, TAP1*; p-value = 1.16e-7).

## DISCUSSION

IFNγ is a cytokine with pleiotropic effects on tumour cells, which is also considered as a crucial mediator of effective antitumour immunity displaying direct impacts on tumour cells [22]. The principal aims of our study were to determine whether IFNγ acts as an epigenetic modifier inducing DNA demethylation and whether the mechanisms by which IFNγ upregulates the expression of selected genes in MHC class I-deficient tumour cells and thus modifies their interactions with the immune system can be associated with DNA demethylation of the corresponding regulatory genes. Reversible MHC class I downregulation on tumour cells can be associated with impaired expression of a number different genes, such as genes encoding MHC class I heavy chains, β2-microglobulin, APM components including LMP-2, LMP-7, TAP-1, TAP-2 and tapasin, which can be coordinated and epigenetically regulated [7,23]. H-2D^b^, H-2K^b^, β2-microglobulin and APM (LMP-2 LMP-7, TAP-1, TAP-2 and tapasin gene expression in our principal model, TC-1/A9, has been analysed previously [15,16,19]. While the expression of genes encoding H-2D^b^, H-2K^b^, β2-microglobulin and tapasin remained robust in TC-1/A9, silencing was observed for LMP-2 LMP-7, TAP-1, TAP-2. Based on these results, we focused on the analysis of the four APM genes that were silenced. However, we cannot exclude that the changes in H-2, β2-microglobulin, tapasin or calnexin expression (and their upregulation upon the IFNγ treatment) can also contribute to the modulation of MHC class I cell-surface expression.

Our data demonstrate that IFNγ-mediated activation of the APM genes and MHC class I expression in two tumour cell lines with reversible MHC class I expression defects was strongly associated with DNA demethylation of multiple APM genes located in the MHC locus. The promoter sequences of the studied APM genes in TC-1/A9 cells were also more methylated as compared to parental TC-1 cells, suggesting that APM gene methylation is involved in MHC class I downregulation on tumour cells that escape from the specific immunity. The data were obtained using four experimental tumour cell lines distinct in origin (transformed by viral oncogenes or by a chemical mutagen) and in metastatic potential.

The finding that the IFNγ-mediated cell signalling can change the methylation status of the promoter regions of multiple genes contributes to our knowledge of the mechanisms underlying regulation of antigen processing and presentation in the context of MHC class I at the transcriptional level. So far, very little is known about the involvement of DNA demethylation in the regulation of multiple genes mediated by the IFNγ signalling pathway. To our knowledge, there is only one study showing that IFNγ-mediated induction of the indole 2,3-dioxygenase (IDO)-1 expression was associated with DNA demethylation of the *IDO-1* gene [24]. Setiadi et al. in 2007 observed that changes in histone acetylation and chromatin remodelling underlie induction of the *TAP-1* expression by IFNγ in TAP-deficient tumour cells [13] and, moreover, massive IFNγ-induced chromatin remodelling of the entire MHC locus, in which both *TAP* and *LMP* genes are located, has been shown by Christova et al. [14]. The MHC genomic region became decondensed, and this was associated with STAT1 phosphorylation followed by binding of the chromatin remodelling enzyme BRG1 at specific sites and subsequent RNA-polymerase II recruitment and histone hyperacetylation, which appeared 2-4 h after the treatment. Our data add to the story and suggest that IFNγ-induced MHC locus chromatin remodelling and histone modifications are associated with DNA demethylation of multiple regions within this genomic locus, resulting in multiple gene expression and increased MHC class I molecule number on the cell surface. Interestingly, the *LMP-7* gene expression was regulated in our experiments in the same manner as other tested APM genes, although the methylation status in the region close upstream to the initiation codon rather remained demethylated. However, demethylation was observed in the more distant region. It is therefore plausible that this gene is controlled by elements located in the wider region of the MHC locus.

Important conclusions can be drawn from the kinetics of these phenomena. DNA demethylation can be either passive, which means dependent on DNA replication when the nascent DNA strains are not methylated due to the DNA methyltransferase deficiency, or active, fast and replication-independent [25]. The data from our kinetic study demonstrated that the DNA demethylation of the *TAP* and *LMP* gene promoter regions was relatively fast, as massive DNA demethylation was seen 6 h after the IFNγ treatments, which roughly corresponded with the kinetics of histone acetylation reported elsewhere and discussed above [14], suggesting an interplay between histone acetylation and DNA demethylation. This is in agreement with the current view on the epigenetic transcription regulation and gene silencing in tumour cells [26]. For comparison, maximum levels of DNA demethylation in the cells treated with DNMTi inhibitor 5-AC were observed 24 after the treatment. This fast kinetics suggests that the demethylation process was active and not dependent on DNA replication, unlike 5AC-mediated demethylation, which requires drug incorporation into DNA and blocks methylation of nascent DNA chains due to methyltransferases inhibition [27]. Interestingly, cytokine-induced DNA demethylation was demonstrated in a study in which TGFβ signalling resulted in active DNA demethylation and p15^ink4b^ tumour suppressor gene expression [28]. We also demonstrated strong association of the DNA demethylation with increased histone acetylation. It is of note that we demonstrate induction of acetylation at histone H3K18, since hypoacetylation at this position has been linked to poor prognosis in several cancers [29].

The data from this and our previous studies [15,18] show that the expression of the studied APM genes in MHC class I-deficient tumours can be increased both by IFNγ and DNA methyltransferase inhibitors. Thus, IFNγ can in some instances serve as a DNA demethylating agent and, on the other hand, DNA methyltransferase inhibitors can upregulate part of the genes that are under the IFNγ signalling pathway control. This means that DNA methyltransferase inhibitors can partially mimic the IFNγ effects on selected immunomodulatory genes, which can be very important for explanation of the immunomodulatory antitumour effects of these compounds.

The data were complemented by a set of transcriptome analyses based on the comparison of cells treated either with a DNMTi DAC, in combination with a histone deacetylase inhibitor TSA or with IFNγ. Two cell lines were included into this experiment: TC-1/A9 is sensitive to both DAC/TSA and IFNγ treatments, when RVP3 cell line is sensitive to DAC/TSA treatment but resistant to IFNγ. Comparative analysis of TC-1/A9 cells treated with either molecules showed a set of 32 genes commonly upregulated (gene set GS-COM) and a set of 73 genes upregulated in IFNγ treatment only (GS-IFN). Both gene sets present significant enrichment for “Cell Death Of Tumour Cell lines”. Interestingly, genes implicated in this term in GS-COM gene set comprise IDO-1 which has already been demonstrated as demethylated by IFNγ in previous studies [24].

GS-COM presents a clear and significant enrichment for antigen-presenting machinery and immunomodulatory genes. It is of importance that the upregulation of APM genes by the epigenetic agents was also seen in an IFNγ non-responding cell line. This represents further evidence demonstrating epigenetic regulation of selected immunomodulatory genes controlled by IFNγ-mediated signalling. Consistent with this finding, the RVP3 cell line (resistant to IFNγ) treated with DAC/TSA, shows a similar list of APM pathway-enriched upregulated genes in common with those found upregulated in IFNγ-treated TC-1/A9 cells. This further confirms the implication of a downstream machinery implicating DNA demethylation for regulating the expression of antigen-presentation molecules triggered by, but not specific to, the IFNγ-transduction cascade.

Collectively, this study documents that IFNγ can act as an epigenetic modifier and induce DNA demethylation of a number of genes, especially those involved in antigen processing and presentation.

## MATERIALS AND METHODS

### Cell lines

MHC class I-positive cell line TC-1 was obtained by *in vitro* co-transfection of murine lung C57BL/6 cells with HPV16 *E6/E7* and activated human *Ha*-*ras* (G12V) oncogenes [30]. The TC-1/A9 (MHC class I-deficient) cell line [19] was obtained from TC-1 tumours developed in immunized mice. Both TC-1 and TC-1/A9 cells were maintained in RPMI 1640 medium supplemented with 10% FCS, 2 mM L-glutamine and antibiotics. E6/E7-expressing MHC class I-deficient cell line MK16/I/IIIABC (MK16), unlike TC-1 and TC-1/A9 metastatic, derived from kidney C57BL/6 cells [31] and murine fibrosarcoma cell line MC15 [32] obtained from methylcholanthrene-treated C57BL/10 mice were maintained under the same conditions as TC-1. The RVP3 cell line [33] was maintained in RPMI 1640 medium supplemented with 10% FCS, 2 mM L-glutamine and antibiotics. The TRAMP-C2 tumour cell line (ATCC collection) was established from a prostate of a PB-Tag C57BL/6 (TRAMP) mouse [34]. The TRAMP-C2 cells were maintained in D-MEM medium supplemented with 5% FCS, Nu-Serum IV (5%; BD Biosciences, Bedford, MA, USA), 0.005 mg/ml bovine insulin (Sigma, St Louis, MO), dehydroisoandrosterone (DHEA, 10 nM; Sigma) and antibiotics. All cell lines were cultured at 37°C in a humidified atmosphere with 5% CO_2_. Cells were cultured in fresh medium for 24 h, after which the medium was removed and the cells were grown in medium containing either rIFNγ (50 U/ml, R&D Systems, Minneapolis, USA ) or 5 μM 5AC (Sigma). Except for the kinetic studies, cells were cultured for 48 h and harvested for analysis.

### Flow cytometry

Cell suspensions were prepared from the cell cultures. Cell surface MHC class I expression on tumour cells was determined using PE anti- H-2D^b^ (clone KH95) and PE anti- H-2K^b^ (AF6-88.5) antibodies. Flow cytometry was performed using an LSR II flow cytometer (BD Biosciences, San Jose, CA), and 10,000 cells were counted. Antibodies used, including the relevant isotypic control, were obtained from Pharmingen, San Diego, CA.

### Real-time quantitative RT-PCR

Total RNA was extracted with RNeasy Mini Kit (Qiagen). The amount of 1 μg of RNA was reverse transcribed to cDNA using random hexamer primers from GeneAmp RNA PCR Core Kit (Applied Biosystems, Foster City, CA) in a 20 μL reaction volume at 42^o^C for 30 min. Quantification of PCR products was performed in 10 μL of Lightcycler 480 SYBR Green I Master mix (Roche) using a real-time PCR Lightcycler (Roche). DNA was denatured at 95^o^C for 2 min; then followed 45 cycles of denaturation at 95^o^C for 25 s, annealing at 60^o^C for 45 s, elongation at 72^o^C for 1 min and incubation at 80^o^C for 5 s. cDNAs were amplified with specific primers for β-actin, *TAP-1, LMP-2, TAP-2,* and *LMP-7*. The list of the *TAP-1, TAP-2, LMP-2*, *LMP-7* and reference genes and their primer sequences have been described elsewhere [15,18]. Fold changes in the transcript levels were calculated using C_T_ values standardized to *β-actin*, used as the endogenous reference gene control. All samples were run in biological triplicates. For statistical analysis of qPCR the Student's t-test was used. Differences between experimental and control samples with P< 0.05 were considered to be statistically significant. The levels of relative gene expression were presented as fold changes compared to the levels found in control samples.

### Bisulphite modification, methylation-specific PCR (MSP) and bisulphite sequencing

Total DNA was extracted with DNeasy Blood & Tissue Kit (Qiagen). Treatment of DNA from TC-1/A9, TC-1, TRAMP-C2 and RVP3 cells with sodium bisulphite and methylation-specific PCR (MSP) analysis of the *TAP-1, TAP-2, LMP-2, LMP-7* promoter regions were performed with Bisulphite Epitect kit (Qiagen, Hilden, Germany) according to the manufacturer's protocol. In order to identify CpG islands within the promoter region of the antigen-processing genes, MSP analysis was performed with primers designed with the program METHPRIMER. The list of the *TAP-1/LMP-2* and *TAP-2* primer sequences has been described elsewhere [15,18]. Two CpG island containing regions within the *LMP-7* upstream sequences were investigated, the primer sequences spanning the more distant to the transcription start have been published previously [15] and the sequences of the primers spanning CpG islands more proximal to the transcription start were as follows: *LMP-7* MSP Un, 5′ TAGGAGGGATATATGAAAAGGTTTG (forward) and AAAATATTAAACAAATCCACCTAAACATA (reverse); *LMP-7* MSP Me, 5′ TAGGAGGGATATATGAAAAGGTTC (forward) and TATTAAACAAATCCACCTAAACGTA (reverse). Within *TAP-2*, upstream sequences were investigated for CpG islands at positions -207 and -214 from the transcription start with forward primer and CpG islands at positions -26 and -40 from the transcription start with reverse primer. Within the *TAP-1/LMP-2* upstream sequences a CpG island was investigated at position +119 from the transcription start with forward primer and CpG islands at positions +278 and +281 from the transcription start with reverse primer. With the *LMP-7* distant primers, CpG islands were investigated at positions -1219, -1233 and -1238 from the transcription start with forward primer and a CpG island at position -1087 from the transcription start with reverse primer. With the *LMP-7* proximal primers, a CpG island was investigated at position -335 from the transcription start with forward primer and CpG islands at positions -186 and -190 from the transcription start with reverse primer. The program for PCR was as follows: 95°C for 2 min, then 35 cycles of 95°C for 2 min, 55°C for 2 min, 73°C for 1:30 min. At the end a final extension period of 73°C for 10 min was added. The PCR products were analysed with gel electrophoresis.

For bisulphite sequencing, another set of primers that amplified both methylated and unmethylated sequences were designed to directly determine the nucleotides resistant to bisulphite conversion. Their sequences were as follows: *TAP-2* BSP, 5′ TTTTGGGTTTAGGTAAGTTTTTTT (forward) and TCTTCTCAAACTAAATCTCCTAAA (reverse); *TAP-1/LMP-2* BSP, 5′ AGTTTTAGGGGTTTTTGATTATTTTAT (forward) and AACTAATAAAACTAACTAAAAATACTA (reverse); *LMP-7* BSP, 5′ GTAGTTTTTGGGTAGATAATGTTT (forward) and AAAACCACAATACCAAAAAAAAA (reverse). Twenty-five CpG islands within the *TAP-2* upstream sequences were investigated, the primer sequences spanning the region from -264 to +76 from the transcription start. Thirty CpG islands within the *TAP-1/LMP-2* upstream sequences were investigated, the primer sequences spanning the region from -335 to +168 from the transcription start. Twenty-five CpG islands within the *LMP-7* upstream sequences were investigated, the primer sequences spanning the region from -502 to +130 from the transcription start. The program for PCR was as follows: 95°C for 5 min, then 25 cycles of 95°C for 50 s, 58°C for 2 min, 72°C for 1:30 min and then 15 cycles of 95°C for 45 s, 54°C for 2 min, 72°C of 1:30 min (+2 s every cycle). At the end, a final extension period of 72°C for 10 min was added. The PCR products were cloned using pGEM®-T Easy Vector System I (Promega). Eleven clones for each of the different DNA sources were sequenced (Applied Biosystem, USA) after thermo-cycle sequencing reaction using the 3.1 version kit.

### JAK/STAT pathway inhibition

JAK Inhibitor I (Cat. No. 420099, Calbiochem) was added into cell cultures 1 h before the IFNγ treatment at a concentration of 2.5 μM. The primary target of JAK Inhibitor I is murine JAK1, its secondary targets are JAK2, JAK3 and TYK2. Fludarabine (Sigma) was used 24 h prior to the IFNγ treatment at a final concentration of 50 μM. Cellular effects of fludarabine were specifically associated with the molecular switch-off of signal transducer and activator of transcription (STAT)-1 activation, without affecting other STAT proteins [35].

### Western blot analysis

Whole-cell protein extracts were prepared from cell line TC-1/A9 using lysis buffer containing 20 mM HEPES (pH 7.9), 150 mM NaCl, 1 mM ethylenediaminetetraacetic acid (EDTA), 1% Triton X-100, 10% glycerol, 1 mM dithiotreitol, and protease or phosphatase inhibitor cocktail tablets (Complete/PhosSTOP, Roche). Proteins (30 μg) were separated in 12% SDS-PAGE gel and transferred to a nitrocellulose membrane (162-0115,Bio-Rad). The membrane was blocked in 0.1% Tris-buffered saline Tween (TBST), 5% skimmed milk for 1 h at room temperature and incubated overnight at 4°C with rabbit polyclonal antibody against STAT1 (Cat. No. 9172S, Cell Signaling) and rabbit monoclonal antibody against the tyrosine-phosphorylated form of STAT1 (Cat. No. 9167S, Cell Signaling). The primary antibody was diluted 1:1000 in TBST containing 5% BSA. After extensive washing, the blot was incubated for 1 h at room temperature with anti-rabbit horseradish peroxidase-conjugated secondary antibody (Cat. No. 7074, Cell Signaling) diluted 1:1000 in TBST containing 5% BSA. After extensive washing of the blots, the bound antibodies were visualized using chemiluminescence (LumiGLO® Reagent, Cell Signaling). GAPDH levels were analysed using rabbit monoclonal antibody (2118S, Cell Signaling; dilution 1:1000) and used as a control for equal loading.

### Chromatin immunoprecipitation assay

Chromatin immunoprecipitation (ChIP) assays were performed as described previously [36] with minor modifications. Briefly, for ChIP experiments, TC-1 or TC-1/A9 cells were grown in 150 cm^2^ culture flasks (TPP) and stimulated with IFNγ (50 U/mL) or left untreated. Two days later the cells were fixed directly in the flasks by addition of 1/10 volume of CRS buffer (11% (v/v) formaldehyde, 100 mM NaCl, 1 mM EDTA, 0.5 mM EGTA, 50 mM HEPES pH 8; Sigma) for 10 min at 4 °C. Cross-linking was stopped by the addition of glycine (final concentration 0.125 M) at 4°C for 5 min. Then, the cells were harvested and resuspended in RIPA buffer (50 mM Hepes, pH 8.0, 150 mM NaCl, 1.0% Triton X-100, 0.1% sodium deoxycholate, 0.1% SDS, 1 mM EDTA, 0.5 mM EGTA; Sigma) containing protease inhibitor cocktail-Complete (Roche), sonicated (Branson 450) and subjected to ChIP analysis. Cell lysate was pre-cleared with 0.05 mg/ml yeast tRNA (Sigma)/protein A/G-agarose beads (Santa Cruz) and then incubated with the rabbit antibody for acetylated histone H3 (Lys18) (Cell Signaling, dilution 1/25). After several washes, DNA bound to the immunocomplexes was obtained and decross-linked by overnight incubation at 65°C. DNA was recovered via phenol/chloroform extraction. The amount of precipitated DNA was analysed using the LightCycler 480 Real-Time PCR System (Roche). Purified (by phenol extraction) input chromatin (chromatin that was not subjected to ChIP) was analysed in control PCR reactions. For the promoter analysis, we designed the following PCR primers which span the *TAP-1/LMP-2* bidirectional promoter: forward GGCAAATCTGCCCAGAGA and reverse CCTAGCCTGGGACTC TCGAC; *TAP-2* promoter: forward CACGGCAGTGAAGTGAAAGC and reverse CAAAAGAACTCACCTGCGGC; *LMP-7* promoter: forward GGACCTAAAGACCCCTGTGC and reverse AGCGGAGGACTGAATAGGGT. The negative control experiments were performed with primers amplifying the gene desert region of DNA: forward CCATGCACATGCTAGCGCTCGA and reverse TCCGAAAGCTGGGAGAAGGGGT.

### Transcriptome analysis

Cell culture protocol published by Suzuki et al. in 2002 [37] was applied with minor modifications. Briefly, for comparative transcriptome analysis, TC-1/A9 and RVP-3 cells were treated either with IFNγ (100 U/ml) for 24 h or with a combination of 3 μM 5-2´-deoxyazacytidine (DAC) for the 48 h (medium was replaced with fresh one with inhibitor after 24 h) and 30 ng/ml Trichostatin A (TSA) for the additional 24 h. RNA was extracted from biological triplicates with RNeasy Mini Kit (Qiagen). The amount of 1 μg of RNA was subjected to the transcriptomic analysis, using Illumina Mouse WG6 bead chips in the Genomic Core Facility at the Institute of Molecular Genetics in Prague. Raw data extraction was performed using Illumina BeadStudio version 2. Raw data analyses were performed using R 3.0.0 software with the packages lumi and limma for data normalization (quantile), transformation (log2) and probe filtering (probes with detection p-values > 0.01 in all samples removed). At least three independently prepared samples (“biological triplicates”) per group were analysed. Statistical analyses were performed using eBayes algorithm with Benjamini-Hochberg p-value correction. Functional annotations were performed using the IPA module from Ingenuity® software. The transcriptome analysis data were deposited at the GEO public functional genomics data repository under the reference Series No. GSE53469.

### SUPPLEMENTAL MATERIAL FIGURE AND TABLES




