# Supplementary material for: Epigenetic regulations in the IFNγ signalling pathway: IFNγ-mediated MHC class I upregulation on tumour cells is associated with DNA demethylation of antigen-presenting machinery genes
Source: Oncotarget. 2014 Jul 15;5(16):6923–35. doi: 10.18632/oncotarget.2222 (PMC4196173; doi:10.18632/oncotarget.2222)
Supplement: Supplementary file 1 [file oncotarget-05-6923-s001.pdf]

Epigenetic regulations in the IFNγ signalling pathway: IFNγ-mediated MHC class I upregulation on tumour cells is associated with DNA demethylation of antigen-presenting machinery genes

Supplementary Material

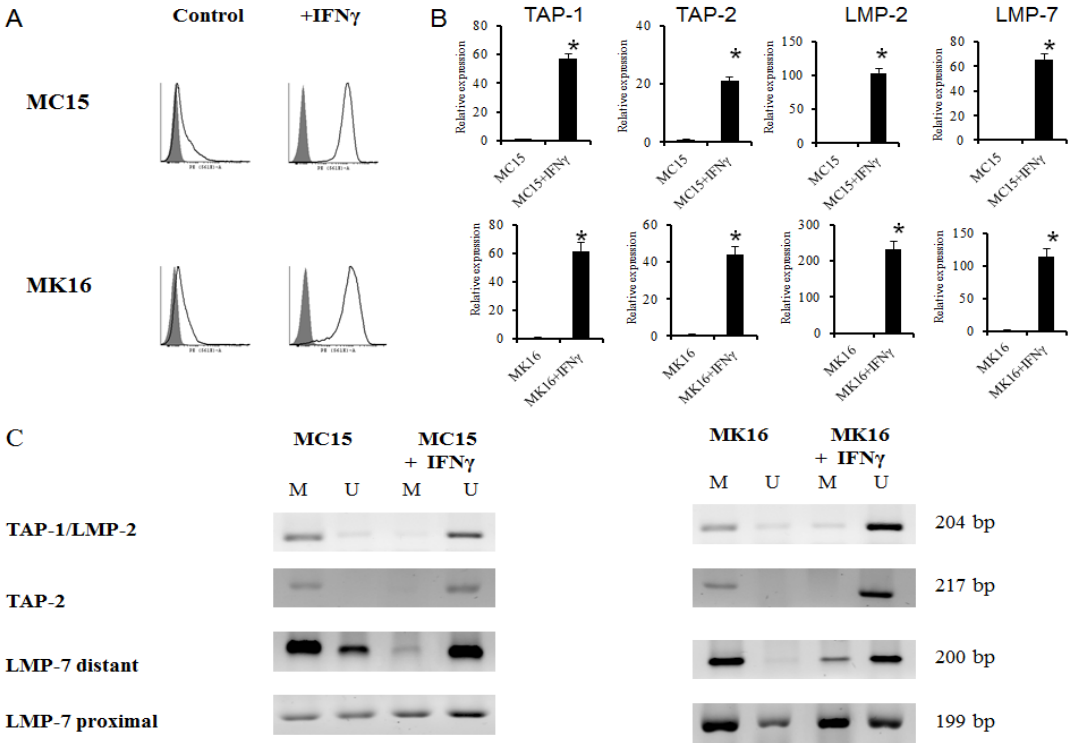

Supplemetary material Figure 1: IFNγ upregulation of the cell-surface MHC class I expression is associated with APM gene expression in experimental MK16 and MC15 tumour cells and stimulates DNA demethylation of the APM gene promoter regions. MHC class I expression (H-2D^{b} and H-2K^{b} together) was determined by the FACS analysis of control tumour cells and after the treatment with IFNγ. Representative results are presented. (A) Upregulation of APM genes in MK16 and MC15 tumours after treatment with IFNγ. (B) Expression levels of selected APM genes in MK16 and MC15 control tumour cells and after the treatment with IFNγ. *denote significant changes ($P<0.05$ determined in Student's t-test) as compared to the values for untreated cells. Biological triplicates were used for the analysis. In all experiments, error bars show standard deviations. Relative expression numbers represent the percentage of the β-actin expression levels. The levels of relative gene expression were presented as fold changes compared to the levels found in control samples.(C) DNA from MK16 and MC15 tumour cell lines cultured in the absence or presence of IFNγ were bisulphite treated and subjected to MSP analysis of the *TAP-1/LMP-2, TAP-2* and *LMP-7* promoter sequences. A higher extent of DNA demethylation induced by IFNγ is documented in MK16 and MC15 cells. U = unmethylated primer, M = methylated. Experiments were repeated two times with similar results.
